# Clinical outcomes of coagulation disorders in elderly Africans: a review of risk, diagnosis, and management

**DOI:** 10.1097/MS9.0000000000004658

**Published:** 2025-12-19

**Authors:** Emmanuel Ifeanyi Obeagu

**Affiliations:** The Division of Molecular Medicine and Haematology, School of Pathology, Faculty of Health Sciences, University of the Witwatersrand, Johannesburg, South Africa

**Keywords:** coagulation disorders, elderly Africans, thromboembolism

## Abstract

Coagulation disorders in the elderly represent a significant source of morbidity and mortality worldwide, with elderly Africans facing unique challenges due to genetic, environmental, and socioeconomic factors. Aging-associated changes in hemostasis, combined with prevalent comorbidities such as infections, cardiovascular disease, and malignancies, increase the susceptibility of this population to thrombotic and bleeding complications. Understanding these risk factors is critical to improving clinical outcomes. Diagnosis of coagulation disorders in elderly Africans is complicated by limited access to specialized laboratory tests, variability in clinical presentations, and overlapping geriatric syndromes. Conventional coagulation assays are often unavailable or unaffordable in many African healthcare settings, leading to delays or inaccuracies in diagnosis. These diagnostic challenges necessitate reliance on clinical acumen and highlight the need for affordable, accessible diagnostic tools adapted to regional contexts.

## Introduction

Coagulation disorders represent a broad spectrum of pathological conditions affecting the delicate balance between clot formation and dissolution, leading to either hemorrhagic or thrombotic complications. These disorders are of particular concern in the elderly population due to age-related physiological changes in the hemostatic system that predispose older adults to an increased risk of thrombosis and bleeding. As the global population ages, the clinical burden of coagulation abnormalities among older adults is rising, warranting focused attention on risk factors, diagnosis, and management tailored to this demographic^[1–3]^. In Africa, the elderly population is growing rapidly as improvements in healthcare extend life expectancy, yet this demographic shift also brings challenges related to chronic diseases, including coagulation disorders. The continent faces a unique set of health disparities, driven by genetic diversity, infectious disease prevalence, socioeconomic factors, and limited healthcare infrastructure. These elements collectively influence the clinical presentation and outcomes of coagulation disorders among elderly Africans, necessitating context-specific understanding and interventions^[4–6]^. The pathophysiology of coagulation disorders in elderly individuals is complex and multifactorial. Aging is associated with increased plasma levels of procoagulant factors such as fibrinogen, factor VIII, and von Willebrand factor, as well as reduced fibrinolytic activity and platelet hyperreactivity. These changes favor a hypercoagulable state, heightening the risk for venous thromboembolism (VTE), stroke, and other thrombotic events. Conversely, age-related vascular fragility and comorbidities can predispose to bleeding complications, further complicating clinical management^[7–9]^.

**HIGHLIGHTS**

- Elderly Africans face heightened thrombotic and bleeding risks due to age-related coagulation changes.
- Limited diagnostic resources hinder the timely identification of coagulation disorders.
- Coexisting conditions complicate management strategies.
- Anticoagulant therapy requires careful monitoring to prevent adverse outcomes.
- Culturally tailored health policies are essential for effective intervention.

Elderly Africans face additional risk factors that exacerbate coagulation abnormalities. High prevalence of infectious diseases such as HIV, tuberculosis, and malaria can trigger disseminated intravascular coagulation (DIC) or alter coagulation pathways. Nutritional deficiencies, including vitamin K deficiency, and genetic predispositions unique to African populations further influence coagulation dynamics. The coexistence of noncommunicable diseases like hypertension, diabetes mellitus, and malignancies amplifies thrombotic risk, often in the setting of limited diagnostic and therapeutic resources^[10,11]^.

Diagnosis of coagulation disorders in elderly Africans is fraught with challenges. Many healthcare facilities lack access to basic coagulation assays such as prothrombin time (PT), activated partial thromboplastin time (aPTT), and D-dimer testing. Advanced diagnostic tools like thromboelastography (TEG) and genetic testing are rare, making comprehensive evaluation difficult. Furthermore, clinical symptoms of coagulation disorders may overlap with other age-related conditions, leading to delayed or missed diagnoses. This diagnostic gap underscores the need for affordable, accessible, and context-appropriate diagnostic strategies^[12–14]^. Management of coagulation disorders in the elderly is inherently complex, requiring individualized balancing of thrombotic and bleeding risks. Anticoagulation therapy is the mainstay for thrombotic disorders; however, elderly patients often have polypharmacy, renal impairment, and frailty that complicate dosing and monitoring. In African settings, challenges include limited availability of direct oral anticoagulants (DOACs), difficulties with regular international normalized ratio (INR) monitoring for warfarin therapy, and poor healthcare follow-up. These factors contribute to suboptimal management and poor clinical outcomes^[15–18]^.

The purpose of this review is to provide a comprehensive synthesis of current evidence regarding coagulation disorders in elderly Africans, with a focus on risk factors, diagnostic challenges, and management strategies. Specifically, we aim to: (1) describe the epidemiology and clinical outcomes of coagulation disorders in this population, (2) identify region-specific risk factors and contributory comorbidities, (3) highlight diagnostic and management challenges in resource-constrained settings, and (4) outline potential strategies to improve patient outcomes. By addressing these gaps, this review seeks to inform clinicians, policymakers, and researchers about the unique challenges of coagulation disorders in Africa’s aging population and to support evidence-based approaches to risk stratification, diagnosis, and care.

## Aim

This narrative review aims to synthesize existing evidence on the clinical outcomes of coagulation disorders in elderly Africans, with a focus on identifying key risk factors, exploring diagnostic challenges, and evaluating current management strategies.

## Epidemiology and risk factors

Coagulation disorders constitute a significant and growing health concern among elderly populations globally, with epidemiological data suggesting an increased incidence of both thrombotic and bleeding events as individuals age. In African countries, the epidemiology of these disorders is less well characterized due to limited surveillance systems and diagnostic capacity, yet emerging studies indicate a substantial burden. VTE, including deep vein thrombosis and pulmonary embolism (PE), represents a leading thrombotic complication in elderly Africans, often presenting with high morbidity and mortality. Additionally, bleeding disorders such as acquired hemophilia and anticoagulant-related hemorrhages are increasingly reported, particularly in the context of anticoagulation therapy^[19–22]^. Aging itself is a primary risk factor for coagulation disorders, as it is associated with significant alterations in the hemostatic system. Elderly individuals typically demonstrate elevated plasma concentrations of procoagulant factors like fibrinogen, factor VIII, and von Willebrand factor, alongside a decline in natural anticoagulants such as protein C and antithrombin. This prothrombotic milieu predisposes them to arterial and venous thrombotic events. Moreover, aging endothelial cells exhibit dysfunction, contributing to a hypercoagulable state. These intrinsic changes are compounded by comorbidities prevalent in the elderly, including atrial fibrillation, hypertension, diabetes mellitus, chronic kidney disease, and cancer, all of which independently increase thrombotic risk^[23–26]^.

In African populations, additional region-specific risk factors influence the epidemiology of coagulation disorders. Infectious diseases endemic to the continent – such as HIV/AIDS, tuberculosis, malaria, and viral hepatitis – play a pivotal role in coagulation abnormalities by triggering systemic inflammation and endothelial injury. For example, HIV infection is associated with both thrombosis and bleeding due to immune activation and opportunistic infections. Nutritional deficiencies, particularly of vitamin K and other micronutrients essential for coagulation factor synthesis, further predispose elderly Africans to bleeding tendencies. Genetic factors also contribute, with certain polymorphisms in coagulation-related genes showing distinct prevalence patterns in African populations, potentially modifying disease susceptibility^[26–29]^. Socioeconomic and healthcare system factors additionally shape the risk profile for coagulation disorders in elderly Africans. Limited access to healthcare services, delayed presentations, and underdiagnosis are common challenges, often resulting in more advanced disease stages and poorer outcomes. Polypharmacy, common in the elderly due to multiple comorbid conditions, increases the risk of adverse drug interactions affecting coagulation status. Moreover, lifestyle factors such as reduced physical activity, malnutrition, and exposure to environmental toxins may also contribute, although these are less well studied in African settings^[30–32]^.

## Recent epidemiological data on aging and coagulation-related morbidity in sub-Saharan Africa

Sub-Saharan Africa is experiencing a demographic transition characterized by a steadily increasing proportion of older adults. Recent estimates indicate that the population aged 60 years and older has grown from approximately 4.9% in 2015 to 6.5% in 2023, with projections suggesting it could reach 8% by 2030. This demographic shift is accompanied by improved life expectancy, rising from 55 years in 2010 to 64 years in 2022, which contributes to a growing burden of age-associated chronic diseases and coagulation disorders^[29]^. Coagulation-related morbidity among elderly Africans remains an under-reported but increasingly recognized health concern. Hospital-based studies across South Africa, Nigeria, Kenya, and Uganda report that VTE affects between 0.5 and 2.3% of hospital admissions in adults over 60 years, often with significant morbidity and mortality. Atrial fibrillation-related ischemic strokes account for approximately 15–20% of strokes in this age group, with anticoagulation therapy underutilized in over 70% of eligible patients^[30]^.

DIC and bleeding disorders complicate 5–10% of severe infections and sepsis cases among older adults, contributing to high in-hospital mortality. The prevalence of comorbid conditions such as hypertension (20–35%) and diabetes (8–12%) further increases thrombotic risk. Additionally, approximately 5–6% of adults over 50 years are living with HIV in sub-Saharan Africa, and infection-associated coagulopathy remains a significant but under-recognized contributor to thrombotic and hemorrhagic complications in the elderly^[31]^. These recent epidemiologic trends underscore the growing importance of understanding coagulation disorders in elderly Africans. They highlight the need for region-specific data, improved diagnostic strategies, and targeted interventions to mitigate morbidity and mortality in this vulnerable population^[32]^.

## Regional insights on coagulation disorders in elderly Africans

### Epidemiology and risk factors

Coagulation disorders among elderly Africans are influenced by a combination of aging physiology, comorbidities, infectious diseases, and environmental factors. Recent studies across sub-Saharan Africa reveal considerable variation in prevalence and presentation. In South Africa, hospital-based surveillance reports that VTE affects approximately 1–2% of elderly admissions, with higher mortality among those with underlying malignancies or cardiovascular disease. In Nigeria, atrial fibrillation-associated stroke is increasingly recognized, accounting for 15–18% of ischemic strokes in adults over 60, yet anticoagulation remains underutilized due to limited access and monitoring challenges^[31]^. Studies from Kenya and Uganda indicate that DIC complicates 5–10% of severe sepsis cases, particularly in the elderly with comorbid infections such as HIV or tuberculosis. Other region-specific risk factors include hypertension, diabetes, and chronic kidney disease, which are prevalent among older adults in countries such as Ghana, Ethiopia, and Tanzania, and contribute significantly to thrombotic risk. Nutritional deficiencies, often associated with socioeconomic constraints, further exacerbate bleeding tendencies in older populations. Collectively, these findings underscore the multifactorial etiology of coagulation disorders in elderly Africans^[32,33]^.

## Clinical outcomes

Clinical outcomes of coagulation disorders vary across regions but are consistently associated with high morbidity and mortality. In South Africa, mortality from VTE-related PE among hospitalized elderly patients ranges from 20 to 25%, highlighting delayed diagnosis and limited prophylactic measures. Stroke outcomes in Nigeria and Ghana are similarly concerning, with 30-day mortality rates approaching 25% among patients with atrial fibrillation. Reports from Kenya, Uganda, and Malawi suggest that elderly patients with infection-related DIC experience in-hospital mortality rates exceeding 40%, particularly when supportive care resources are limited^[34]^.

## Diagnostic and management challenges

Across the continent, diagnostic capacity remains a major barrier. Many facilities lack access to coagulation assays, imaging modalities, and standardized reference ranges tailored to African populations. In rural Tanzania and Zambia, reliance on clinical assessment alone often delays detection of thrombotic or hemorrhagic events. Management challenges are similarly compounded by the limited availability of anticoagulants, inconsistent blood product supply, and inadequate monitoring infrastructure. In South Africa, DOACs are available but remain prohibitively expensive for most elderly patients, while warfarin therapy requires regular INR monitoring, which is often inaccessible outside urban centers^[35]^.

## Diagnostic challenges

Accurate and timely diagnosis of coagulation disorders in elderly Africans presents significant challenges that directly impact clinical outcomes. One of the foremost obstacles is the limited availability of laboratory infrastructure and diagnostic resources in many African healthcare settings. Basic coagulation screening tests such as PT, aPTT, platelet counts, and D-dimer assays are often unavailable or inconsistently accessible, particularly in rural and resource-limited areas. This scarcity hampers the ability of clinicians to confirm suspected coagulation abnormalities and differentiate between thrombotic and bleeding disorders effectively^[33,34]^. In addition to resource constraints, there is a notable lack of standardized reference ranges for coagulation tests that consider age, ethnicity, and local population characteristics. Many African laboratories rely on reference values derived from Western populations, which may not accurately reflect physiological variations in African elderly patients. This discordance can lead to misinterpretation of results, either underestimating or overestimating the severity of coagulation abnormalities. Moreover, age-related physiological changes can alter coagulation test results, complicating the differentiation between normal aging processes and pathological coagulation disorders^[35,36]^.

Clinical presentation of coagulation disorders in the elderly may also be atypical or nonspecific, often overlapping with other common geriatric syndromes such as anemia, infections, or chronic inflammatory states. Symptoms like bruising, fatigue, or edema may be attributed to other chronic diseases, resulting in delayed suspicion and diagnosis. Cognitive impairment and communication difficulties frequently encountered in elderly patients further complicate history-taking and symptom reporting^[37]^. Advanced diagnostic modalities such as TEG, rotational thromboelastometry, and genetic assays for thrombophilia are largely unavailable in many African health facilities due to cost and technical expertise requirements. The absence of these tools limits the comprehensive assessment of coagulation dynamics and hereditary risk factors. Consequently, clinicians often rely heavily on clinical acumen and empirical treatment, which may be suboptimal^[38]^. Furthermore, the coexistence of infectious and noncommunicable diseases can confound laboratory results. For example, systemic infections such as sepsis or malaria can induce DIC, altering coagulation profiles and masking underlying chronic coagulation disorders. HIV-associated coagulopathies present additional diagnostic complexity, as antiretroviral therapy and opportunistic infections can influence coagulation parameters (Table 1)^[39,40]^.

**Table 1:** Diagnostic challenges and proposed solutions for coagulation disorders in elderly Africans

Diagnostic challenge	Description/impact	Proposed solutions
Limited laboratory infrastructure	Many rural and district hospitals lack coagulation analyzers and imaging modalities (Doppler ultrasound, CT angiography), delaying diagnosis	Implement point-of-care (POC) coagulation testing; prioritize essential tests in tiered laboratory algorithms
Inadequate reference ranges	Reliance on non-African population reference intervals can lead to misinterpretation of coagulation parameters	Develop standardized, population-specific and age-specific reference ranges for African populations
Delayed clinical recognition	Healthcare personnel may have limited exposure to geriatric-specific hematologic disorders, leading to late diagnosis	Targeted training programs, workshops, and telemedicine mentorship to improve recognition of coagulation abnormalities
Resource constraints for follow-up testing	Repeated testing is often unavailable, limiting longitudinal monitoring of thrombotic or bleeding disorders	Simplified monitoring protocols, integration of clinical risk scores (e.g., Caprini, Padua), and POC follow-up assessments
Limited diagnostic integration	Fragmented systems make it difficult to combine laboratory, imaging, and clinical data for timely decision making	Establish integrated care pathways and standardized diagnostic algorithms suitable for low-resource settings
High prevalence of comorbidities	Coexisting infections, malnutrition, or chronic diseases may confound coagulation test interpretation	Incorporate comorbidity-adjusted risk assessment tools and multidisciplinary evaluation into diagnostic workflows

## Management strategies

Effective management of coagulation disorders in elderly Africans requires a comprehensive and individualized approach that balances the risks of thrombosis and bleeding while addressing the underlying causes. Anticoagulation remains the cornerstone of treatment for thrombotic conditions such as VTE, atrial fibrillation, and stroke prevention. However, management in elderly patients is inherently challenging due to age-related changes in pharmacodynamics and pharmacokinetics, polypharmacy, comorbidities, and increased susceptibility to bleeding complications^[41]^. In many African healthcare settings, warfarin remains the most accessible oral anticoagulant due to cost constraints, despite its narrow therapeutic window and need for regular INR monitoring. Unfortunately, routine INR testing is often unavailable or irregular, leading to suboptimal dosing and increased risks of both hemorrhagic and thrombotic events. DOACs offer several advantages, including fixed dosing and no routine monitoring; however, their high cost and limited availability restrict their use in most African countries. These barriers necessitate pragmatic approaches, including close clinical monitoring and patient education to enhance adherence and early identification of complications^[42,43]^.

Management of bleeding disorders, whether acquired or inherited, also presents distinct challenges. Supportive therapies such as transfusion of clotting factors, platelets, or fresh frozen plasma may be required but are frequently constrained by availability and cost. In conditions like acquired hemophilia or DIC, treatment of the underlying precipitating factors – such as infections, malignancies, or medication-induced coagulopathy – is critical to restoring hemostatic balance^[44]^. Addressing comorbidities that contribute to coagulation abnormalities is essential. Control of hypertension, diabetes, and infections such as HIV and tuberculosis reduces thrombotic risk. Nutritional support, including vitamin K supplementation when deficiencies are suspected, is an important adjunct. Rehabilitation and lifestyle modifications aimed at increasing mobility can further decrease venous stasis and associated thrombotic events^[45]^. Healthcare infrastructure improvements are vital to optimize management outcomes. Establishing accessible coagulation monitoring services, training healthcare providers in geriatric anticoagulation management, and developing region-specific clinical guidelines can improve treatment safety and efficacy. Community education campaigns to raise awareness about symptoms of thrombosis and bleeding, as well as adherence to therapy, can empower patients and caregivers to participate actively in care (Table 2)^[46,47]^.

**Table 2:** Management strategies for coagulation disorders in elderly Africans

Management challenge	Description/impact	Proposed strategies
Anticoagulation therapy access	Limited availability and high cost of DOACs; warfarin requires regular INR monitoring, often inaccessible in rural areas	Utilize context-appropriate anticoagulants; implement simplified dosing and monitoring protocols; explore government or NGO-supported drug programs
Bleeding management	Elderly patients may experience gastrointestinal, intracranial, or post-procedural bleeding, exacerbated by comorbidities and malnutrition	Ensure availability of blood products; use vitamin K and hemostatic agents as indicated; monitor high-risk patients closely
Management of DIC	High mortality due to infection-associated or malignancy-related DIC; supportive care is limited in many facilities	Early recognition through POC testing; treat underlying cause promptly; provide supportive transfusions and fluids as needed
Multimorbidity complicating therapy	Coexisting conditions (e.g., HIV, hypertension, diabetes) increase thrombotic and bleeding risk	Individualized treatment plans; multidisciplinary teams including physicians, hematologists, and infectious disease specialists; comorbidity-adjusted risk assessment tools
Resource-limited monitoring	Limited capacity for repeated laboratory tests or imaging; challenges in longitudinal follow-up	Integrate clinical risk scores with simplified laboratory follow-up; use POC devices for dynamic monitoring; community-based follow-up where feasible
Patient education and adherence	Lack of awareness of anticoagulation importance and therapy adherence issues	Provide culturally appropriate patient education; counseling on adherence, diet, and monitoring; community health worker support

## Conclusion

Coagulation disorders represent a growing and under-recognized health challenge among elderly Africans, driven by the interplay of aging physiology, comorbidities, infectious diseases, and resource-limited healthcare settings. Current evidence highlights substantial morbidity and mortality associated with VTE, atrial fibrillation-related stroke, DIC, and bleeding disorders, yet region-specific data remain limited. Addressing these challenges requires a multifaceted approach. Strengthening diagnostic capacity through point-of-care testing, simplified laboratory algorithms, and locally validated reference ranges can facilitate early detection. Expanding healthcare worker training and implementing context-specific clinical guidelines are essential to improve recognition and management of coagulation disorders. In addition, enhancing access to anticoagulation therapies, establishing national registries, and integrating risk assessment tools can guide preventive and therapeutic strategies.

Future research should focus on generating robust, Africa-specific epidemiologic data, evaluating cost-effective interventions, and developing scalable models of care tailored to elderly populations in diverse African settings. By adopting these strategies, clinicians and policymakers can reduce preventable morbidity and mortality, improve patient outcomes, and strengthen healthcare systems to meet the needs of Africa’s aging population.
